# Antigenic, Immunologic and Genetic Characterization of Rough Strains *B.abortus* RB51, *B.melitensis* B115 and *B.melitensis* B18

**DOI:** 10.1371/journal.pone.0024073

**Published:** 2011-10-31

**Authors:** Rosanna Adone, Michele Muscillo, Giuseppina La Rosa, Massimiliano Francia, Michela Tarantino

**Affiliations:** 1 Dipartimento Sanità Pubblica Veterinaria e Sicurezza Alimentare, Istituto Superiore di Sanità, Roma, Italy; 2 Dipartimento Ambiente e Connessa Prevenzione Primaria, Istituto Superiore di Sanità, Roma, Italy; Universite Libre de Bruxelles, Belgium

## Abstract

The lipopolysaccharide (LPS) is considered the major virulent factor in Brucella spp. Several genes have been identified involved in the synthesis of the three LPS components: lipid A, core and O-PS. Usually, Brucella strains devoid of O-PS (rough mutants) are less virulent than the wild type and do not induce undesirable interfering antibodies. Such of them proved to be protective against brucellosis in mice. Because of these favorable features, rough strains have been considered potential brucellosis vaccines. In this study, we evaluated the antigenic, immunologic and genetic characteristics of rough strains B.abortus RB51, B.melitensis B115 and B.melitensis B18. RB51 derived from B.abortus 2308 virulent strain and B115 is a natural rough strain in which the O-PS is present in the cytoplasm. B18 is a rough rifampin-resistan mutant isolated in our laboratory.

The surface antigenicity of RB51, B115 and B18 was evaluated by testing their ability to bind antibodies induced by rough or smooth Brucella strains. The antibody response induced by each strain was evaluated in rabbits. Twenty-one genes, involved in the LPS-synthesis, were sequenced and compared with the B.melitensis 16M strain.

The results indicated that RB51, B115 and B18 have differences in antigenicity, immunologic and genetic properties. Particularly, in B115 a nonsense mutation was detected in wzm gene, which could explain the intracellular localization of O-PS in this strain.

Complementation studies to evaluate the precise role of each mutation in affecting Brucella morphology and its virulence, could provide useful information for the assessment of new, attenuated vaccines for brucellosis.

## Introduction

In *Brucella* spp., as in many other gram-negative bacteria, the smooth lipopolysaccharide (S-LPS) is an important component of the outer membrane, strongly involved in pathogenesis mechanisms. Its precise role as a virulence factor is not yet clear. It has been suggested, however, that the LPS molecule may play a key role in the invasion and intracellular multiplication of *Brucella* spp. as well as in protecting the cell against complement-mediated lysis. Moreover, the LPS is the immunodominant antigen to which the majority of antibodies resulting from either infection or vaccination are directed [1]–[4].

The S-LPS molecule has three sections: the lipid A, the core oligosaccharide and the distal O-polysaccharide chain (O-PS or O-antigen). The O-PS is a homopolymer of N-formyl-perosamine.

*Brucella* strains carrying complete S-LPS have a smooth (S) phenotype, so termed after the smooth texture of the colonial surface, while *Brucellae* devoid of O-PS have a rough (R) phenotype.

*B.abortus*, *B.melitensis*, *B.suis*, *B.neotomae*, *B.microti*, as well as the recently isolated *B.pinnipedialis* and *B.ceti* species, express a smooth phenotype, while *B.abortus* RB51, *B.melitensis* B115, *B.ovis* and *B.canis* are typically rough strains [5], [6].

Smooth-to-rough phase variation can spontaneously occur in *Brucella* smooth strains as result of environmental factors but the molecular mechanism responsible for such variation has not yet been defined [7]–[9]. Owing to the lack of antigenic O-PS, true R-mutants neither induce anti O-PS antibodies which could interfere with a serologic diagnosis of brucellosis, nor react with anti-O-PS antibodies [5], [10]. In addition, these mutants show outer membrane morphological and physiological changes resulting in the uptake of crystal violet and the autoagglutination in acriflavine solution [5]. With the exception of *B.ovis* and *B.canis*, which are rough but virulent, R-mutants are less virulent than the wild type.

Because of these features, *Brucella* R-mutants have been considered potential brucellosis vaccines [11], [12]. The strain RB51 has replaced the S19 as vaccine for brucellosis in cattle in many countries [12]. RB51 is a spontaneous R-mutant derived from the virulent strain *B.abortus* 2308 after a series of passages in selective media [10]. It expresses no O-PS on its cell surface, and therefore induces no diagnostically undesirable antibodies, mainly directed against this antigen [10], [13], [14]. Nevertheless, it produces anti-RB51 antibodies, as detected by specific serologic tests [13], [15]. Genetic analysis showed that RB51 carries the genetic element IS*711*, spontaneously inserted into the *wboA* gene [16]. Complementation of RB51 with *wboA*, has been shown to increase O-PS expression without, however, restoring the smooth phenotype, suggesting the presence of additional mutations responsible for its rough morphology [17]–[20].

*B.melitensis* B115 is a natural, stable, rough strain, the phenotype of which has been evaluated according to classical criteria [5]. Many reports confirmed the lack of surface O-PS. Additional studies, however, demonstrated the presence of detectable O-antigen in the cytoplasm [21], [22].

The mechanism of LPS synthesis is largely unknown, but genetic studies indicate that it is similar to that existing in some gram-negative bacteria. Several genes have been proven to be involved in the biosynthetic pathways of lipid A, core, and O-PS [7], [11], [16], [19], [23], [24]. Most of these genes are clustered in two genetic regions, *wbk* and *wbo*. R-mutants can result from mutations affecting O-PS precursor synthesis, its polymerization and export, or from a variety of defects in the inner core polysaccharide [11], [25], [26].

In preliminary studies conducted in our laboratory to assess the potential value of B115 as vaccine, B115 was unable to induce antibodies to O-PS in mice, even when administered at high dosages. Nevertheless, it elicited specific anti-B115 antibodies and was able to confer good protection [27], [28]. *B.melitensis* 18 is a rough, stable, rifampin-resitant mutant of *B.melitensis* isolated in our laboratory by several passages on *Brucella* agar medium supplemented with rifampin. B18 showed different antigenic and immunological properties compared to other strains – despite its rough morphology, it induced detectable anti-O-PS antibodies in laboratory animals [29].

In this study, we compared the antigenic and immunologic characteristics of RB51, B115 and B18. A molecular analysis, for each strain, of the 21 genes known to be involved in LPS synthesis was also performed. The genes of the three rough strains under study were PCR-amplified and sequenced, and the results compared to a smooth reference strain *B.melitensis* 16M the sequences of which had been published in GenBank [30]. This preliminary study would ultimately allow an in-depth investigation of potential correlations between immunologic characteristics and genetic makeup, necessary for the development of novel *Brucella* vaccines.

## Materials and Methods

### Bacterial strains and growth conditions

*B.melitensis* B115 and *B.abortus* 99 were provided to us by the Veterinary Laboratories Agency (VLA) of Weybridge (U.K.), while the *B.abortus* RB51 vaccine strain was isolated from the commercial vaccine “RB-51 CZV”, (Cooper-Zeltia Veterinaria, SA, Spain). *B.melitensis* B18 was isolated in our laboratory after several passages on *Brucella* agar medium supplemented with rifampin, as previously described [29]. All *Brucella* strains were cultured at 37°C for 48 h on Trypticase Soy Agar (TSA, OXOID) supplemented with 5% sterile horse serum.

### Animals

For serological analysis, New Zealand white (NZW) rabbits, weighting 1.2–1.5 kg, provided by the Harlan Italy (Udine, Italy), were used. All animal experiments were conducted in ABL3 facilities, in keeping with current European legislation (directive 86/609/EEC).

### Ethics statement

Experiments with mice, according to National (D.L. 116/92) and European (86/609/EEC) regulations, were previously authorized by the National Authority (Decreto 225/2009-B). Bovine serum samples collected from *B.abortus* 99-naturally-infected cattle were provided by the Istituti Zooprofilattici Sperimentali, responsible for the serological surveillance of herds as prescribed by the Italian brucellosis eradication plan. For these sera, no ethical approval was sought, as they were obtained from animals that contracted the infection naturally.

### Antigenic characterization and antibody response evaluation

The smooth or rough phenotype of *Brucella* strains was determined by classical criteria, such as crystal violet staining, acriflavine agglutination (0.1% w/v) and agglutination with monospecific *Brucella* sera (to A and M antigens) [5]. Cell-surface expression of O-PS was tested by evaluating the ability of each strain to bind antibodies induced by smooth or rough *Brucella* strains, in Complement Fixation Tests (CFTs). Bacterial suspensions were prepared with whole cells of *B.abortus* 99 smooth strain, *B.melitensis* B115, *B.abortus* RB51 and *B.melitensis* B18. All bacterial strains were grown at 37°C on TSA, adjusted to a concentration of 10^9^ CFU/ml (OD_600_ = 0.170) and then heat-inactivated. Before their use as antigen in CFT, an equal amount of fetal calf serum (FCS, GIBCO) was added to RB51 suspensions as previously described [13], [15]. This was done in order to neutralize possible nonspecific reactions due to the anticomplementary activity characteristic of rough phenotype brucella strains. B18 required the same treatment to avoid the anti-complementary activity. In contrast, *B.melitensis* B115 was used as is, since it showed no anticomplementary activity in a previous study [27]. To evaluate antibody response to *Brucella* strains, three groups of five NZW rabbits were inoculated subcutaneously (s.c.) with 10^9^ CFU/rabbit of viable RB51, B18 and B115, respectively. Blood samples were collected by heart puncture at 7, 15, 30, 60, 90, and 140 postinoculation days (PID). Blood was allowed to clot for 12 hours at 4°C and then centrifuged. Serum samples were divided into 1 ml aliquots and stored at −20°C until use.

The presence of anti-O-PS antibodies was evaluated by the CFT and the Rose Bengal Plate Test (RBPT), using *B.abortus* 99 smooth strain as antigen, according to Alton et al. [5]. To detect antibodies directed against antigens other than O-PS, the CFT was performed using B115 as rough antigen, as previously described [27]. Anti-OPS antibodies were obtained from serum samples taken from *B.abortus* 99-naturally-infected cattle, provided to our laboratory by the Istituti Zooprofilattici Sperimentali. Antibodies directed against antigens other than O-PS were obtained from serum samples collected from RB51-vaccinated cattle, previously provided by Dr. Olsen (United States Department of Agriculture Ames, IA, USA) for a collaborative study to validate a specific serologic test [13]. CFTs were performed in microtitre U-bottom plates as described elsewhere [13], [15], [27].

### Genetic characterization of *Brucella* strains: DNA preparation, PCR assay and sequence analysis

*Brucella* strains were cultured for 48 h at 37°C on TSA slopes. The pellet was resuspended in 467 µl TE/sodium buffer (50 mM Tris, 50 mM EDTA, 100 mM NaCl, pH 8) and digested for 1 hr at 37°C with 3 µl of 20 mg/ml proteinase K and 30 µl of 10% SDS. DNA was purified twice with phenol-chloroform using Phase Lock Gel Heavy tubes (Eppendorf AG, Hamburg, Germany). Nucleic acids were precipitated using isopropanol/sodium acetate and the pellet resuspended in 50 µl of nuclease-free water. A total of 21 genes involved in LPS synthesis (17 located in chromosome I and 4 in chromosome II) were analyzed for each *Brucella* strain. The *Brucella* genes under study and the primers used for PCR amplifications, designed using the Primer3 software (http://frodo.wi.mit.edu/cgi-bin/primer3/primer3_www.cgi), are listed in Table 1. A PostgreSQL database was previously created to keep track of all primers, PCRs and samples, each of which was assigned an identification code (ID). A convenient web interface allows internet connection to this database, available to registered users at https://cosmos.bio.uniroma1.it. PCR amplifications were carried out using the TripleMaster PCR System and the High Fidelity Buffer (Eppendorf AG, Hamburg, Germany) following the suggested protocol for high fidelity PCR. About 100 ng of genomic DNA were used as template for each reaction. Thirty amplification cycles were performed, each comprising denaturation at 95°C for 2 min, annealing at 55°C for 1 min, and extension at 72°C for 1 min. Ten µl of each reaction mixture were analyzed by electrophoresis on a 2% agarose gel stained with ethidium bromide.

**Table 1:** Primers used for PCR and sequencing analysis of genes, involved in the LPS synthesis, examined in this study.

PCR	Primers	Sequence (5′→3′)	Specificity (gene)	Locus^*^	Putative role**	Amplicon size (bp)
**347**	1153	AGTGGACGAAACTTGGGATG	*wboB*	BMEI 0997	Mannosyltransferase	1686
	1154	ATCTCTGCATTCGTGGGAAG				
**348**	1156	GGGTTATAGCCGATAAACACG	*wboA*	BMEI 0998	Glycosyltranferase	1297 (2139 in RB51)
	1157	CCCGCGCATTAAGAGTAGAC				
**349**	1159	ATGAGCGGACCATAAAGGTG	*wa* 1326	BMEI 1326	protein of unknown function	2253
	1160	GCGCTGATTGAGGAAAAGAC				
**350**	1162	ATGCCTCATCTACGCTCCAT	*wbkE*	BMEI 1393	Glycosyltranferase	1287
	1163	TGTCGAATTTTTGGTTGTCG				
**351**	1165	CGACAACCAAAAATTCGACA	*manA_OAg_*	BMEI 1394	Perosamine synthesis	1256
	1166	GAGGATGATTTCGGACGTGT				
**352**	1168	GACAACCAAAGAGGCAAAGC	*manB_OAg_*	BMEI 1395	Perosamine synthesis	1592
	1169	TGCAAAGCTCTCTGAAACGA				
**353**	1171	GCGGATGAATTCAGATCGTT	*manC_OAg_*	BMEI 1396	Perosamine synthesis	1466
	1172	GTGCCATCCATCATGTGAAC				
**354**	1174	CCTCCTTGCTATCCGATTGA	*wbkA*	BMEI 1404	Glycosyltranferase	1271
	1175	ATTTGCAAAATCTGGGCAAC				
**355**	1177	TGTAATGCTCCACGAGCAAG	*gmd*	BMEI 1413	Perosamine synthesis	1257
	1178	ACCCTGTATCAAGGCATTCG				
**356**	1180	GCTGGAAGCCGAAAATCTCT	*per*	BMEI 1414	Perosamine synthesis	1297
	1181	CACCAGAAGTGGCGTACCTT				
**357**	1183	AGCGCTTCCACAGGATCTTA	*wzm*	BMEI 1415	ABC transporter permease	995
	1184	ATGCAGCGTAGTGCAGATGA				
**358**	1186	TGCTTAGGTGTGAGCCTCCT	*wzt*	BMEI 1416	ABC transporter permease	915
	1187	AGAGGGACCTCACATTCTCG				
**359**	1189	AACTCGGGAATGGGAGCTAT	*wbkB*	BMEI 1417	Unknown	934
	1190	CAAAACGCGTGTATTTGGTG				
**360**	1192	CGTTACCCAAGTTGGAATGC	*wbkC*	BMEI 1418	Formyltranferase	890
	1193	CTGCTGGGACCAAAAACCTA				
**361**	1195	GTTAGCGGAGGAATGGACAA	*wbkF* (ex *wecA*)	BMEI 1426	Undecaprenyl-P-α-N-acetylglucosaminyl	1213
	1196	CGCCATGGAAAAGAAGGATA				
**362**	1198	CCACACTCATTCTGCTGCTC	*wbkD*	BMEI 1427	Unknown	2042
	1199	GGTCTGTTACGGTTGCTGGT				
**363**	1201	CACTTGCTGCCTTGATTGAG	*pgm*	BMEI 1886	Phosphoglucomutase	1956
	1202	TCACTGCCCTACTGCCCTAC				
**364**	1204	ACAGGAGAATTTTCGGCGTA	*wa* 0053	BMEII 0053	Transport ATPase	976
	1205	ATCGCTGTTGCAACCTACCT				
**366**	1210	GGCAGATACAGGTTCGATGG	*manB-core*	BMEII 0899	Phosphomannomutase	1577
	1211	GGTCTTGCCGATGAGTGTTT				
**367**	1213	TGGAATCTTCGGATGACACA	*manC-core*	BMEII 0900	Unknown	1526
	1214	TTCGCATTTGGCTGAATATG				
**368**	1216	GATGTGGGACTTCCGTTCTG	wa****	BMEII 1134	Amidase (?)	1290

GeneBank AE008917 (chromosome I) and AE008918 (chromosome II);

**Moriyon *et al.* (2004).

Following amplification, PCR products were purified using a Montage PCRm96 Micro-well Filter Plate (Millipore, Billerica, Mass). Bidirectional sequencing was performed using the amplification primers listed in Table 1. Purified products were sequenced by Macrogen Inc. (10F World Meridian Center 60-24 Kumchun-Ku, Kasan-Dong, Seoul, Korea 153-023) using an ABI3730 XL DNA Analyzer (Applied Biosystems, Renton, USA). The forward and reverse ABI files were aligned and assembled into a single consensus sequence using MEGA software version 5 (http://www.megasoftware.net/). All sequences were submitted to BLAST analysis (http://blast.ncbi.nlm.nih.gov/Blast.cgi/) and nucleotide and amino acid changes identified through comparison with the corresponding *B.melitensis* 16M sequences available in GenBank (AE008917 for chromosome I and AE008918 for chromosome II).

## Results

### Surface antigenicity and antibody response in rabbits

The rough phenotype of RB51, B115 and B18, tested by the classical methods of crystal violet staining and autoagglutination reaction with acriflavine, was confirmed after passages in culture media. No reversion to smooth phenotype occurred after passages in mice and rabbits (data not shown).

In CFTs performed on whole-cell antigen suspensions of RB51, B18 and B115, *B.abortus* 99 smooth strain, tested as control, bound only anti-O-PS antibodies. RB51 and B115 bound antibodies induced by the rough strain RB51, and did not react with anti-OPS antibodies induced by the smooth strain *B.abortus* 99.

In contrast, the rough strain B18 was able to react with both kinds of antibodies – anti-O-PS and anti-RB51 - giving the same titres obtained with homologous antigens used separately.

The antibody response induced by *Brucella* strains in rabbits was evaluated by CFTs and RBPT, up to 140 PIDs. As expected, vaccination with RB51 did not induce antibodies to O-PS. At PID 7, 80% of rabbits had seroconverted to the rough antigen B115, reaching 100% at PID 15. Specific antibodies were still present in 60% of RB51-inoculated rabbits at PID 140.

Similarly, no antibodies to O-PS were detected in sera from rabbits inoculated with B115. Antibodies to B115 were detected in 80% of these rabbits at PID 7, and in 100% at PID 15. At PID 140, 80% of rabbits were still seropositive.

In the group vaccinated with B18, 80% of rabbits had reacted with both antigens by PID 7, giving similar titres. By PID 15, both kinds of antibodies had appeared in 100% of the animals, that had remained seropositive until PID 90. At pid 140, however, anti-O-PS antibodies were detected in 80% of rabbits, while only 40% had antibodies to B115.

### Molecular analysis

The results of the molecular analysis performed on 21 genes involved in LPS synthesis are presented in Table 2. Consensus sequences of B115, RB51 and B18 strains were compared to the available *B. melitensis* 16M genome (AE008917 for chromosome I and AE008918 for chromosome II) in order to detect mutations. The presence of each mutation was confirmed after passages in mice, thus confirming the genetic stability of mutants (data not shown).

**Table 2:** Mutations detected in LPS-synthesis genes of *B.melitensis* B115, *B.abortus* RB51 and *B.melitensis* B18.

PCR	Gene	D	*B.melitensis* B115			*B. abortus* RB51			*B.melitensis* B 18		
			nucleotide position	protein position	T	nucleotide position	protein position	T	nucleotide position	protein position	T
**347**	*wboB* (1560 bp)	←	None			T to C (347)	L to S (116)	M	T to C (347)	L to S (116)	M
						T to C (447)	G to G (149)	S			
						A to G (690)	A to A (230)	S			
						T to C (756)	Y to Y (252)	S			
						G to A (971)	R to Q (324)	M			
**348**	*wboA* (1233 bp)	←	None			842 bp insertion(706)	frameshift (236)	N	C to A (943)	H to N (315)	M
									T to G (1086)	S to S (362)	S
**349**	*wa -1326* (2166 bp)	→	None			C to T (278)	S to L (93)	M	C to T (278)	S to L (93)	M
									T to G (651)	A to A (217)	S
									A to C (801)	Q to H (267)	M
**350**	*wbkE* (1134 bp)	←	None			C to T (216)	L to L (72)	S	None		
**351**	*manA_Aog_* (1164 bp)	←	None			None			None		
**352**	*manB_Oag_* (1425 bp)	←	None			C to T (1141)	L to F (381)	M	G to A (210)	A to A (70)	S
									C to T (1141)	L to F (381)	M
**353**	*manC_Oag_* (1374 bp)	←	A to G (903)	R to R (301)	S	A to G (395)	D to G (132)	M	T to C (397)	F to L (133)	M
						T to C (397)	F to L (133)	M			
						C to T (817)	L to F (273)	M	A to G (903)	R to R (301)	S
						A to G (903)	R to R (301)	S			
**354**	*wbkA* (1119 bp)	←	None			C to A (1047)	N to K (349)	M	None		
						T to C (1059)	S to S (353)	S			
**355**	*gmd* (1089 bp)	→	None			A to G (519)	E to E (173)	S	None		
						G to C (975)	P to P (325)	S			
**356**	*per* (1143 bp)	→	C to T (690)	Y to Y (230)	S	T to C (144)	S to S (48)	S	None		
**357**	*wzm* (798 bp)	→	G to A (236)	W to STOP (79)	N	C to A (360)T to C (455)	V to V (120)L to P (152)	SM	None		
**358**	*wzt* (759 bp)	→	None			A to C (512)	D to A (171)	M	None		
**359**	*wbkB* (855 bp)	→	None			None			None		
**360**	*wbkC* (780 bp)	→	None			None			None		
**361**	*wbkF* (1008 bp)	→	None			G to A (48)	G to G (16)	S	None		
**362**	*wbkD* (1869)	←	None			C to T (263)	T to I (88)	M	A to C (615)	P to P (205)	S
						G to A (1300)	V to I (434)	M	C to T ( 920)	T to M (307)	M
						A to G (1338)	K to K (446)	S	A to G (1338)	K to K (446)	S
						G to A (1675)	E to K (559)	M	G to A (1757)	G to D ( 586)	M
						A to G (1819)	K to E (607)	M	A to G (1819)	K to E (607)	M
**363**	*pgm* (1701 bp)	→	A to G (752)	H to R (251)	M	A to G (752)	H to R (251)	M	A to G (752)	H to R (251)	M
									C to T (1527)	S to S (509)	S
**364**	*wa* (0053) (741 bp)	←	None			T to C (672)	S to S (224)	S	None		
**366**	*manBcore* (1434 bp)	→	C to T (279)	G to G (93)	S	C to T (279)	G to G (93)	S	C to T (279)	G to G (93)	S
			C to T (1335)	R to R (445)	S						
**367**	*manCcore* (1416 bp)	→	None			G to C (64)	D to H (22)	M	None		
**368**	wa **** (1206 bp)	→	None			T to A (388)	S to T (130)	M	A to C (751)	S to R (251)	M
						A to C (751)	S to R (251)	M			
						T to C (758)	I to T (253)	M	C to T (1155)	S to S (385)	S

D: direction of transcription of the gene; T: type of mutation; M: missense; S: silent; N: nonsense. Mutations identified are referenced to *B.melitensis* 16M.

(AE 008917 for chromosome I and AE008918 for chromosome II).

Of the 21 genes examined, three showed no nucleotide mutations compared to the genomic sequences of strain 16M (*manA_OAg_*, *wbkB*, *wbKc*) in any of the three strains analyzed. Six genes presented only silent mutations, with no resulting change to the amino acid sequence of the corresponding protein (*wbkE*, *gmd*, *per*, *wbkF*, *wa0053*, *manBcore*). Most genes showed a number of missense mutations (*wboB*, *wboA*, *wa-1326*, *manB_OAg_*, *manC_OAg_*, *wzt*, *wzm*, *wbkD*, *pgm*, *manCcore*, and wa****). Two nonsense mutations were also detected, one already known, that is, the interruption of the *wboA* gene by an IS711 element in *B. abortus* RB51; the other, a point mutation at position 236 in the *wzm* gene for the B115 strain, that changes the amino acid tryptophan (W) into a stop codon in position 79 of the protein, resulting in a polypeptide chain that ends prematurely and a truncated protein product.

## Discussion

In the present study, we evaluated the antigenic, immunologic and genetic characteristics of rough strains *B.abortus* RB51, *B.melitensis* B115 and *B.melitensis* B18. We amplified and sequenced 21 genes involved in LPS synthesis, in order to detect genetic mutations which could ultimately be correlated with the different properties of these strains. All sequences were compared with those of *B.melitensis* 16M reference strain.

RB51 and B115 are well known strains. Their antigenic, genetic and immunological characteristics, however, have not been fully assessed. B18 is a rough, rifampin-resistant mutant of *B.melitensis* isolated in our laboratory [29].

In *Brucella* strains of smooth phenotype, LPS is synthesized as two separate components, lipid A-core and the O-PS. The O-PS is a homopolymer of N-formyl-perosamine units [24], [31]. Several genes involved in LPS biosynthesis have been recognized, most of them clustered in the *wbk* and *wbo* genetic regions [11]. The *wbo* region encodes two putative glycosyltranferases, *wboA* and *wboB*.

The w*bk* contains genes coding for enzymes necessary for N-formylperosamine synthesis (*gmd*, *per*), its formylation (*wbkC*) and polymerization (*wbkE*, *wbkA*), for bactoprenol priming (*wbkD* and *wbkF*) and the ABC transporters for the O-PS translocation (*wzm*, *wzt*). It also contains genes involved in the synthesis of mannose (*manA_OAg_*, *manB_OAg_* and *manC_OAg_*), which probably act coordinately with *gmd* and *per*, and independently with other genes [18], [32], [33]. Finally, three genes have been shown to be involved in core synthesis - pgm, manBcore and wa**** [26].

These genetic regions, central for LPS synthesis in smooth strains, may be expected to be absent or different in rough strains. And indeed, *B.ovis* is naturally devoid of the wbo region [34]. The *wbk* region, however, is present both in *B.ovis* and in *B.canis*, which are natural rough strains [25]. The disruption of *wbkB* and *manBOAg* does not generate rough mutants [24], [26].

Transposon mutagenesis and complementation studies, generating different rough mutants, confirmed the crucial role of these genes [24], [26].

*B.abortus* rough strain RB51 has no O-PS exposed on its cell surface but it is not completely devoid of O-PS: it has been demonstrated that RB51 produces low levels of M-like O-antigen [35]. Genetic analysis revealed that RB51 carries an IS*711* element spontaneously inserted into the *wbo*A gene, which maps outside of the main *wbk* region [16]. The *wbo*A encodes for a glycosyltranferase that is essential for the polymerization of the O-antigen [17]: disruption of this gene in *B.abortus*, *B.melitensis* and *B.suis* resulted in rough mutants that were unable to synthetize the O-PS [36]. The complementation of RB51 with functional *wbo*A (RB51WboA) increased O-PS expression, but the resulting low levels of O-PS were present in the cytoplasm, and not on the surface [19], [20]. It has been suggested that other LPS biosynthetic genes may have been modified affecting the export of O-PS and S-LPS to the bacterial surface or the appropriate coupling of the O-antigen to the core-LPS or both. In mice, the RB51WboA strain induced detectable antibodies to the O-antigen and showed an enhanced vaccine efficacy against the *B.abortus* 2308 virulent strain, without affecting its attenuation characteristic [19], [20].

*B.melitensis* B115 is a natural rough strain, whose phenotype has been found to be very stable, even after passages *in vivo* [5], [37]. Based on its *rpoB* nucleotide sequence, it has been classified as *B.melitensis* biotype 1 [38]. Many studies confirmed the absence of surface O-PS in this strain. Interestingly, however, low-titre anti-O-PS antibodies have been detected in rabbits immunized with live or killed B115 [37] and O-PS specific monoclonal antibodies obtained when mice were immunized with B115 [39], [40]. Immunogold labelling using O-PS specific monoclonal antibodies revealed that the O-PS was present in the cytoplasm and that some of the expressed O-antigen was lipid-bound and associated with the cell wall [21], [22]. In previous studies conducted in our laboratory, however, B115 didn't induce antibodies to O-PS in mice, even when it was given twice at high dosages, while detectable anti-B115 antibodies were produced [27], [28].

Several hypotheses have been formulated to explain the rough phenotype of *B.melitensis* B115 despite its O-chain expression [39]. Two genes, *wzm* and *wzt*, have been identified as encoding proteins with high similarity to several two-component ABC transporters. Their involvement in O-PS translocation across the inner membrane was confirmed by gene replacement. The deletion of these two genes resulted in an inability to express LPS. *B.melitensis wzm/wzt* mutants, generated in *B.melitensis* 16M by allelic replacement strategy, resulted in colonies of rough phenotype with intracellular accumulation of free O-PS, not bound to the lipid A-core molecule [24], [41].

When functional *wzm* and *wzt* genes cloned into plasmids were supplied, the wild-type phenotype was restored.

In contrast, in B115, the same complementation failed to restore the smooth phenotype. The possibility has, thus, been raised, that additional mutations may be present, affecting the LPS O-side-chain transport or ligation to the lipid A- core molecule [24].

In this study, we tested the surface O-PS expression of RB51, B115 and B18 by evaluating their ability to bind antibodies directed against O-PS or against antigens other than O-PS. This was done by CFTs, using whole-cell suspensions of RB51, B115 and B18 as antigens.

CFT results confirmed that RB51 whole cells bind homologous antibodies and fail to react with antibodies directed to O-PS. Similarly, whole cells of B115 did not bind anti-O-PS antibodies, as previously indicated [42], but did react with antibodies induced by the rough strain RB51. Interestingly, however, B18 was able to bind both kinds of antibodies.

Moreover, like RB51, the strain B18 needed to be previously incubated with FCS to neutralize anticomplementary activity causing non specific reactions in CFTs. In contrast, B115 was naturally devoid of anticomplementary activity and did not require the addition of FCS.

Our data thus confirm that, despite the rough phenotype in common, the three strains under study - RB51, B115 and B18 - present differences in their surface antigenicity, including the expression of O-PS on the cell-surface of the B18 strain.

The antibody response induced by RB51, B18 and B115 was tested in three groups of rabbits, respectively. Antibodies to O-PS were detected using the *B.abortus* S99-based CFT [5], while specific antibodies induced by rough *Brucella* strains were detected by a B115-based CFT [43].

The CFT was used as a serological test because of its ability to detect antibodies induced by rough *Brucella* strains with satisfactory sensitivity and specificity, as previously demonstrated [13], [27].

The results confirmed that B115 and RB51 fail to elicit anti-O-PS antibodies, while both induce specific antibodies still detectable at PID 140 . The lack of anti-O-PS antibodies in B115 and RB51-inoculated rabbits was confirmed by *B.abortus* 99-based RBPT. The production of specific antibodies induced by B115 and RB51 in rabbits was similar to that observed in mice in a previous study [27].

All B18-inoculated rabbits produced both anti-O-PS antibodies and specific anti-B18 antibodies, in similar titres. At PID 140, 80% of sera still contained low levels of antibodies to O-PS, while specific anti-B18 antibodies were detected only in 40% of rabbits. This may have been due to a relatively low sensitivity of the B115-based CFT in detecting anti-B18 antibodies.

Serological results confirmed the absence of surface O-PS in RB51 and B115 and the presence of the O-PS antigen in B18. We didn't investigate the presence of intracellular O-PS in B18. Our antigenic results, however, suggest that it may be exposed at surface.

These data also indicate that intracellular O-PS in B115 is incapable of eliciting detectable O-PS response. This is not surprising; a similar phenomenon has been reported for the Δ*pgm* rough mutant, which harbours small amounts of cytoplasmic O-antigen but fails to induce antibodies to O-PS in mice [4]. The nature of this lack of response remains to be determined.

The genetic analysis of RB51, B115 and B18, performed by comparing the sequences of 21 genes involved in LPS-synthesis with those of the *B.melitensis* 16M strain, indicated the presence of some mutations, most of them missense mutations.

The nucleotide and amino acid differences detected in the genes examined are shown in Table 2. Passages in mice confirmed the stability of all mutations.

Sequence analysis of B115 genes revealed high homology with *B.melitensis* 16M, with few genetic differences detected - six point mutations, four of the silent type, one missense, and one nonsense. Silent mutations were present in genes *manC_OAg_*, *per* and *manBcore*. A missense mutation was detected in the *pgm* gene. This mutation was also present in RB51 and B18. The gene *pgm* plays a central role in O-PS biosynthesis since it codes for the enzyme phosphoglucomutase which is responsible for the interconversion of glucose-6-phosphate to glucose-1-phosphate, and is thus essential to the biosynthesis of glucose or galactose in *B.abortus* [3].

A nonsense mutation was detected in the *wzm* gene. Since this gene was found to be responsible for the incorporation of the O-PS to the periplasmic side of the membrane, such mutation may be linked to the rough morphology of B115. In addition, this mutation - detected only in B115 - can therefore be used as a marker for the molecular identification and differentiation of B115.

In RB51, beyond the well known insertion in gene *wboA* [16], [17], we detected several genetic differences between the RB51 strain and the published *B.melitensis* 16M genome (Table 2). Silent mutations were detected in *wbkE*, *gmd*, *per*, *wbkF*, *wa* (0053) and *manBcore*. Missense mutations were detected in eleven genes: *wboB*, *wa*-1326, *manB_OAg_*, *manC_OAg_*, *wbkA*, *wzm*, *wzt*, *wbkD*, *pgm*, *manCcore* and wa****. The significance of these mutations and the correlation with the rough morphology are not easy to interpret, as not all missense mutations lead to protein changes able to disrupt normal function.

The mutations detected in genes *wzm* and *wzt* could explain the fact, that while complementation of RB51 with a functional *wboA* increases intracellular O-chain expression, it does not restore the smooth phenotype. As shown, some of the mutations identified in RB51 were also present in B18.

In B18, missense mutations were detected in eight of the 21 genes sequenced. The genes showing this kind of mutation were *wboB, wboA, wa-1326, manB_OAg_*, manC_OAg_, wbkD, pgm and wa**** Only one silent mutation was detected, in *manBcore*, while the sequences of genes *wbkE*, *manA_OAg_*, *wbkA*, *gmd*, *per*, *wzm*, *wzt*, *wbkB* and *wbkC*, *wbkF*, *wa*(0053), *manCcore* were identical to those of the *B.melitensis* 16M reference strain.

Interestingly, in strain B18, which seemed to express surface O-PS, genes *wbkA*, *gmd*, *per*, *wzm*, *wzt*, *wbkB* and *wbkC* showed no genetic diversity.

These genes had been identified as involved in the biosynthesis of the LPS O-side chain [25]. It has later been shown, however, that they were highly conserved not only in the classical S-type *Brucella* species, but also in the rough strains *B.ovis* and *B.canis*, suggesting that other genes should be responsible for the rough morphology [25].

Our results indicate that the rough strains RB51, B115 and B18, characterized by different antigenic and immunologic properties, show differences in genes involved in LPS synthesis. We were able to identify the specific genes affected by such mutations. These findings may provide a valuable basis for further investigations (e.g. complementation studies) to assess the precise role of each mutation in determining the *Brucella* phenotype and its characteristics, essential for the development of attenuated and immunogenic vaccines.
